# Safety and efficacy of autologous non-hematopoietic enriched stem cell nebulization in COVID-19 patients: a randomized clinical trial, Abu Dhabi 2020

**DOI:** 10.1186/s41231-021-00101-5

**Published:** 2021-11-03

**Authors:** Yendry Ventura-Carmenate, Fatima Mohammed Alkaabi, Yandy Marx Castillo-Aleman, Carlos Agustin Villegas-Valverde, Yasmine Maher Ahmed, Pierdanilo Sanna, Ayesha Abdulla Almarzooqi, Abeer Abdelrazik, Gina Marcela Torres-Zambrano, Maura Wade-Mateo, David Quesada-Saliba, Loubna Abdel Hadi, Antonio Alfonso Bencomo-Hernandez, Rene Antonio Rivero-Jimenez

**Affiliations:** 1 Abu Dhabi Stem Cells Center, Al Misahah Street, Villa No. 25, Rowdhat, Zone-1, POB 4600, Abu Dhabi City, United Arab Emirates; 2 Sheikh Khalifa Medical City Hospital, POB 51900, Abu Dhabi City, United Arab Emirates; 3 McHari International College, Nassau, Bahamas; 4 Miami Dade College, Mathematics Department Chair, Wolfson Campus, Miami, FL 33132 USA

**Keywords:** COVID-19, Stem cells, Nebulizers and vaporizers, Immunomodulation, Recovery of function

## Abstract

**Background:**

The novel SARS-CoV-2 has caused the coronavirus disease 2019 (COVID-19) pandemic. Currently, with insufficient worldwide vaccination rates, identifying treatment solutions to reduce the impact of the virus is urgently needed.

**Method:**

An adaptive, multicentric, open-label, and randomized controlled phase I/II clinical trial entitled the “SENTAD-COVID Study” was conducted by the *Abu Dhabi Stem Cells Center* under exceptional conditional approval by the Emirates Institutional Review Board (IRB) for COVID-19 Research Committee from April 4th to July 31st, 2020, using an autologous peripheral blood non-hematopoietic enriched stem cell cocktail (PB-NHESC-C) administered by compressor (jet) nebulization as a complement to standard care therapy. The primary endpoints include safety and efficacy assessments, adverse events, the mortality rate within 28 days, and the time to clinical improvement as measured by a 2-point reduction on a seven-category ordinal scale or discharge from the hospital whichever occurred first.

**Results:**

The study included a total of 139 randomized COVID-19 patients, with 69 in the experimental group and 70 in the control group (standard care). Overall survival was 94.20% for the cocktail-treated group vs. 90.27% for the control group. Adverse events were reported in 50 (72.46%) patients receiving PB-NHESC-C and 51 (72.85%) in the control group (*p* = 0.9590), with signs and symptoms commonly found in COVID-19. After the first 9 days of the intervention, 67.3% of cocktail-treated patients recovered and were released from hospitals compared to 53.1% (RR = 0.84; 95% CI, 0.56–1.28) in the control group. Improvement, i.e., at least a 2-point reduction in the severity scale, was more frequently observed in cocktail-treated patients (42.0%) than in controls (17.0%) (RR = 0.69; 95% CI, 0.56–0.88).

**Conclusions:**

Cocktail treatment improved clinical outcomes without increasing adverse events. Thus, the nebulization of PB-NHESC-C was safe and effective for treatment in most of these patients.

**Trial registration:**

ClinicalTrials.gov. NCT04473170. It was retrospectively registered on July 16th, 2020.

## Introduction

In December 2019, a novel member of the family of severe acute respiratory syndrome coronaviruses named SARS-CoV-2 rapidly spread from China throughout the globe, causing a pandemic of the human respiratory illness recognized as coronavirus disease 2019 (COVID-19) [1]. The United Arab Emirates (UAE) has shown a sharp and chaotic increase in the number of confirmed COVID-19 patients [2] despite the country’s nationwide efforts to control the disease by introducing a series of strict measures to stop the spread of infection. After a noticeable surge of cases, the UAE declared a state of emergency to fight coronavirus, emphasizing the need for a new approach for treatment to decrease disease progression and related deaths.

SARS-CoV-2 infection can cause a broad spectrum of symptoms, ranging from mild to severe illness. COVID-19 severe cases are characterized by severe pneumonia, acute respiratory distress syndrome (ARDS), excessive acute inflammatory responses, development of cytokine storms, and multiple organ failures leading to death. In contrast, non-severe cases present known clinical manifestations of respiratory system infection [3, 4]. Although clinical aspects of COVID-19 patients have been widely reported and their management has increased tremendously, safe and effective medications are still lacking. Indeed, several clinical trials have been developed and are still under investigation for COVID-19 prevention, treatment, and diagnosis [5–7].

Cellular therapy, a new field of medicine that uses cell-based products, is considered a pillar of regenerative and personalized medicine [8]. Different types of stem cells, including embryonic stem cells and adult stem cells, such as hematopoietic stem cells (HSCs), mesenchymal stem cells (MSCs), induced pluripotent stem cells (iPSCs), and, more recently, very small embryonic-like stem cells (VSELs), have been considered “candidates” in several therapies [9, 10]. Adult stem cells are somatic, rare, and undifferentiated populations located in their niches within several adult solid organs and play a key role in maintaining tissue homeostasis. Additionally, several studies have reported that adult stem cells, including VSELs, are self-renewing and multipotent, exist in a dormant state, and are recruited to the peripheral blood (PB) under an alarm recall to support the role of innate immunity [11, 12]. Accordingly, adult stem cells are most likely present at high concentrations in the bloodstream of COVID-19 patients in response to exuberant inflammatory processes. However, good immune phenotyping of stem cells in the PB after SARS-CoV-2 infection is needed to develop potential cell therapeutic applications. To date, MSCs have shown a potential therapeutic effect in COVID-19. Several clinical trials have been registered for MSC-based therapies using different routes for delivery, including intravenous, intratracheal instillation, and inhalation/nebulization [13, 14].

Unfortunately, the challenge in adult stem cell therapy is the lack of well-established procedures for stem cell characterization, including cell isolation, purification, and expansion, without altering their phenotypes and functions [15]. To overcome these challenges, our group has developed a patented method for isolating an autologous peripheral blood non-hematopoietic enriched stem cell cocktail (PB-NHESC-C) [16] to be administered by the nebulization route.

The objective of this study was to assess the safety and efficacy of PB-NHESC-C stem cell cocktail nebulization as an add-on therapy to standard care for SARS-CoV-2-infected patients during the COVID-19 outbreak in Abu Dhabi, UAE.

## Methods

### Participants and study design

The safety and efficacy of an autologous PB-NHESC-C in patients with COVID-19 were evaluated by an adaptive, multicentric, open-label, and randomized controlled phase I/II clinical trial called the “SENTAD-COVID Study”. The Abu Dhabi Stem Cells Center (ADSCC) designed the study protocol and received exceptional conditional approval by the Ministry of Health and Prevention via the Emirates Institutional Review Board (IRB) for the COVID-19 Research Committee. It was performed in Abu Dhabi from April 4th to July 31st, 2020, using procedures based on ethical standards of the Helsinki Declaration of 1975 (as revised in 1983), such as informed consent for all participants. Patients of at least 15 different nationalities who resided in the emirate of Abu Dhabi were hospitalized and treated in four major government hospitals, with *Sheikh Khalifa Medical City Hospital* serving as the main primary care clinical trial unit. The study’s sample size was established trying to detect a clinically significant difference between both groups concerning hospital stay and mortality with 80% power and a 5% alpha error. Permuted block randomization was used to allocate patients to the treatment groups. Each “block” had a specified number of randomly ordered treatment assignments.

#### Patients inclusion and exclusion criteria

**Fig. 1:**
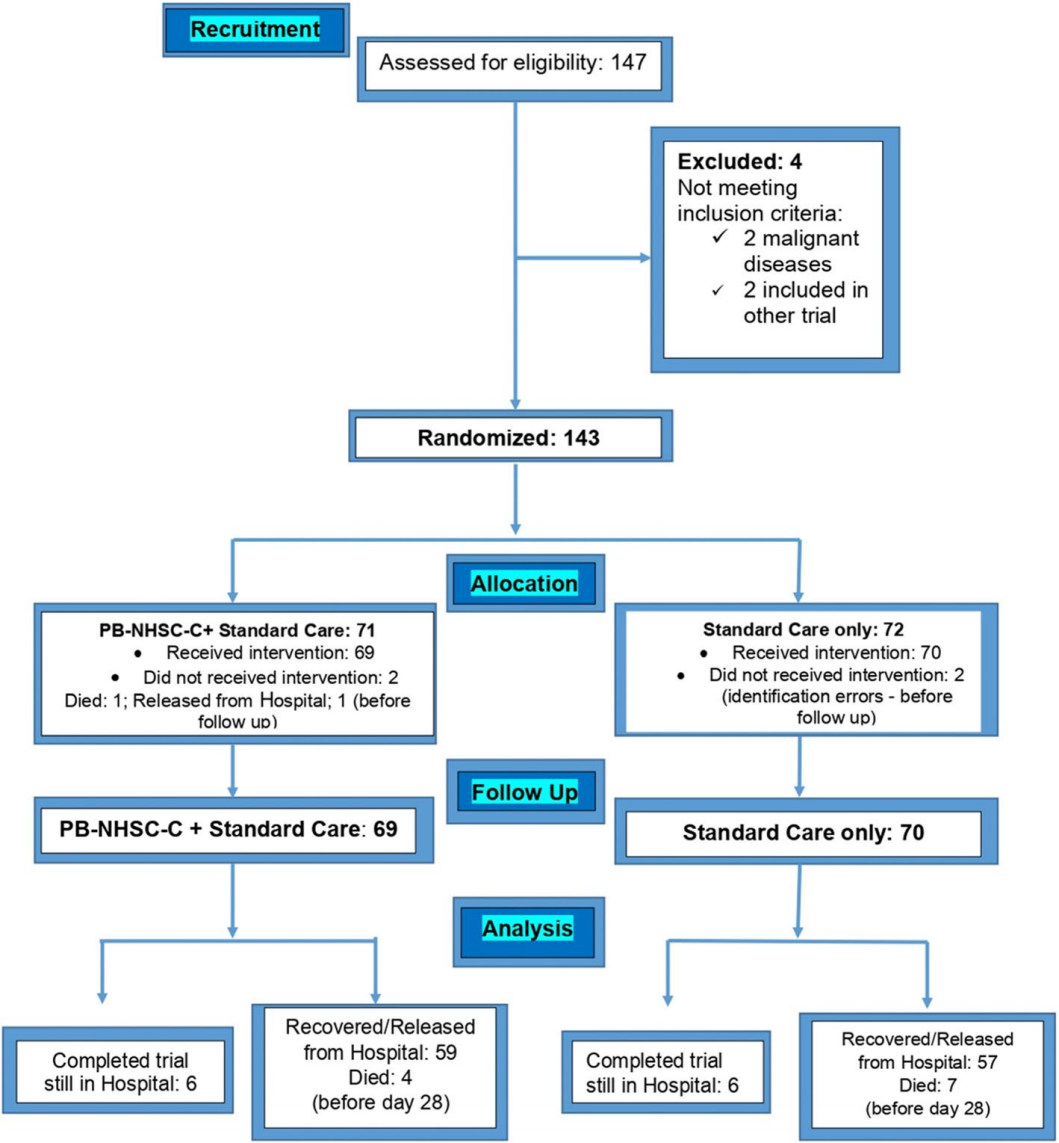
Patient allocation during the SENTAD-COVID Study. Legend: Four initially screened patients were excluded before randomization because 2 had been previously diagnosed with malignant diseases, and 2 were previously included in other clinical trials. After randomization, data from 4 additional patients were not analyzed. 2 patients in the control group had data error identifications. 2 patients were not followed in the PB-NHESC-C group: 1 was released from the hospital, and the other unfortunately died before the initial day of treatment

Inclusion: Real-Time Polymerase Chain Reaction (RT-PCR) Laboratory confirmation of COVID-19; male or female aged ≥18 years; Hospitalized and symptomatic patients, referring one or more of the following symptoms (fever, cough, or shortness of breath), in association with (at least one): tiredness, runny nose, headache, sore throat, chills, muscle pain, or new loss of taste or smell; Ability to comply with test requirements and for peripheral blood stem cells collection. Exclusion: Pediatric patients (aged < 18 years); Diagnosis of any shock; Organ transplants in the past 3 months; Patients receiving immunosuppressive therapy; Diagnostic of Hepatitis B Virus (HBV) infection; Diagnostic of Human Immunodeficiency Virus (HIV) infection or Acquired Immunodeficiency Syndrome (AIDS); Current diagnosis of cancer; History of malignancies in the past 5 years; Pregnant or lactating women; Having participated in other clinical trials in the past 3 months; Inability to provide informed consent. On the clinical site, selected investigators/physicians in charge of patient care communicated with the trial subjects. When patients met the inclusion/exclusion criteria, they were recruited after they or their legal representative signed the informed consent. A doctoral supervisor approved collecting data from clinical records and an initial clinical evaluation. A total of 139 COVID-19 patients with HCoV-19 RNA confirmed by RT-PCR assay [17] were randomly included, as shown in Fig. 1.

Finally, patients were categorized according to their disease severity and clinical manifestations at the recruitment based on guidelines established by the *interim guidance* of the World Health Organization (WHO) as follows: scores of 1–2 represent patients in ambulatory conditions (1 indicates a completely asymptomatic patient, and 2 represents a patient with some limitation of activity); scores of 3–4 represent hospitalized patients with mild disease (3 indicates a hospitalized patient without oxygen therapy, and 4 indicates a patient receiving oxygen by mass or nasal prongs); scores of 5–7 represent patients hospitalized with severe disease (5 represents a patient with no invasive ventilation or high flow oxygen, 6 represents a patient with intubation and mechanical ventilation, and 7 represents a patient under ventilation plus additional support, including vasopressor, renal replacement therapy, or extracorporeal membrane oxygenation); a score of 8 indicates a deceased patient [18].

### PB-NHESC-C: preparation, and characterization

#### Preparation of the investigational product

Briefly, autologous PB-NHESC-C was prepared in a closed system using a 300 mL collected of PB in quadruple blood bags (Haemonetics®, WBT436CCA, MA, USA). First, the bags were centrifuged in a Hettich centrifuge (Roto Silenta 630 RS, Tuttlingen, FRG) at 800×g to obtain platelet and stem cells rich plasma. Next, centrifugation separated the platelet-concentrated plasma (PCP) from the stem cells. Finally, PCP was used to produce platelet-derived growth factor (PDGF) by sonication. Stem cells were suspended in 30 mL of PDGF to obtain the cocktail and stored at 4–8 °C for 24 h before clinical application, which could be performed within 5 days after its preparation [16]. The final investigational product keeps viability between 90 and 95% up to 10 days stored at 4–8 °C**.**

#### Flow Cytometry analysis

**Fig. 2:**
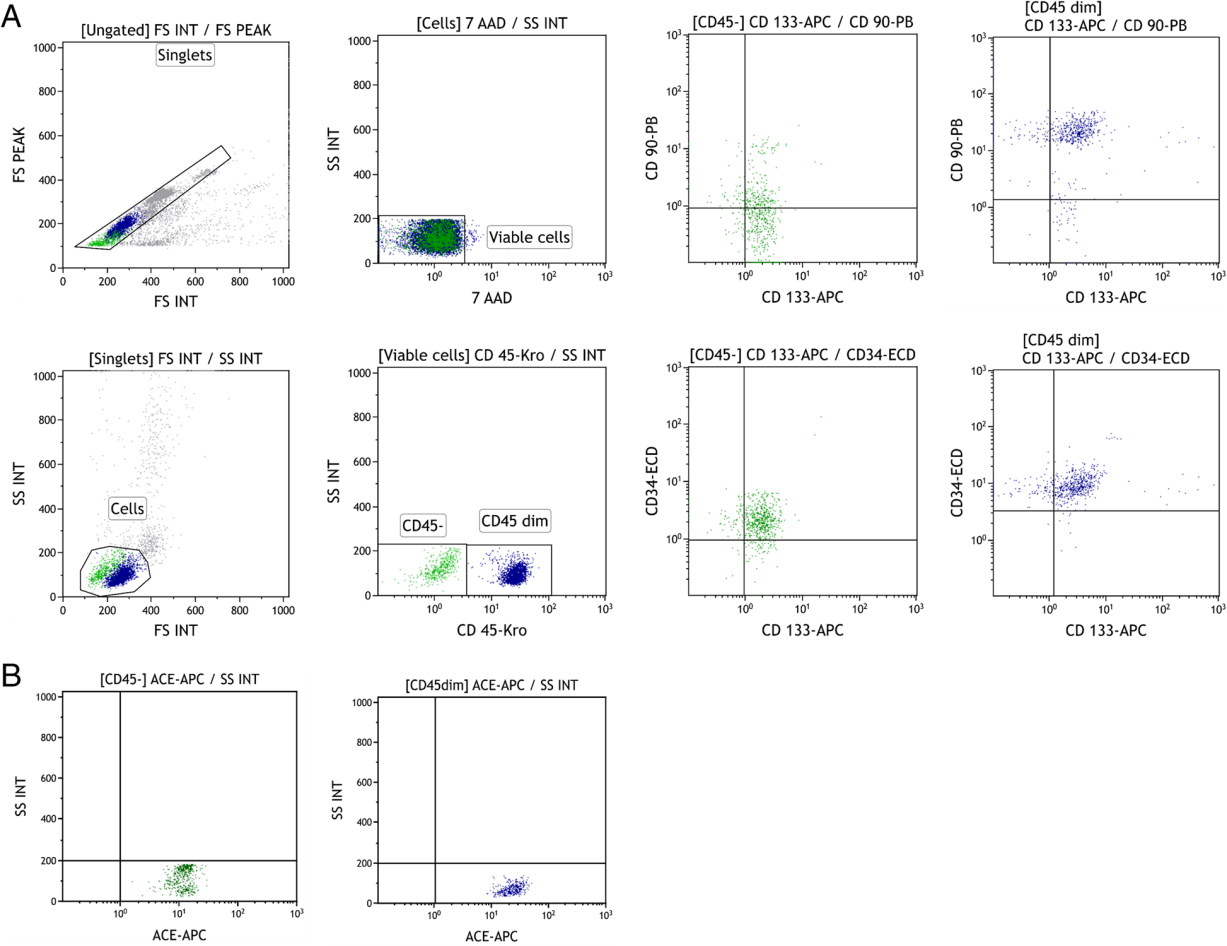
Flow cytometry gating strategy. Legend: Immunophenotype characterization of peripheral blood non-hematopoietic enriched stem cell cocktail. 2A) Logic and manual gating strategy for cell characterization using five monoclonal antibody-conjugated CD markers simultaneously, including 7-Amino-Actinomycin D (7-AAD). 2B) Expression of angiotensin-converting enzyme 2 (ACE2)

The stem cells cocktail was characterized by identifying hematopoietic stem cells subsets expressing CD34^+^, CD133^+^, CD90^+^, CD45^+^, and non-hematopoietic stem cells identified as CD45 negative using a Navios EX flow cytometer (Beckman Coulter, USA). Then, 100 μL of PB-NHESC-C was dispensed for staining with fluorochrome-conjugated monoclonal antibodies from the same manufacturer: Krome Orange (Kro) anti-CD45 (Kro, clone J33); Pacific-blue (Pb) anti-CD90 (Pb, clone Thy-1/310); Allophycocyanin (APC) anti-CD133 (APC, clone W6B3C1); and R-Phycoerythrin-Texas Red®-X (ECD) anti-CD34 (ECD, clone 581). Cell viability was evaluated with 7-amino actinomycin D (7-AAD) to exclude dead cells from analysis. After 30 min of incubation at room temperature (RT) in the dark, cells were treated for 10 min with 500 μL of OptiLyse C. Before an immediate running, 100 μL flow-count fluorospheres were added for absolute cell counts. Angiotensin-Converting Enzyme 2 (ACE2) Surface Expression Determination: 100 μL of PB-NHESC-C were incubated for 20 min in the dark with 20 μL of the anti-CD45 (Kro; clone J33), 20 μL of 7-AAD, and 5 μL of anti-CD143 (APC; clone 5–369, Biolegend). Acquisition data were processed using Kaluza C Software V1.1 with a minimum of 150,000 acquired events. The gating strategy is shown in Fig. 2A.

#### Immunofluorescence analysis

Samples of PB-NHESC-Cs were fixed with 3.5% paraformaldehyde for 20 min, pre-blocked with 2% bovine serum albumin for 10 min at RT, and subsequently stained with fluorescein isothiocyanate (FITC)-conjugated CD45 (1:100, mouse monoclonal IgG; Beckman Coulter) for 30 min at RT in the dark. Hoechst 33342 nucleic acid stain (Sigma Aldrich) was added at 10 μg/mL to the cell suspensions for 20 min at RT in the dark. After washing, PB-NHESC-C was acquired using a laser scanning microscope (Leica SP8 confocal microscope, Leica) with FITC (emission 496–598 nm) and Hoechst (emission 415–470 nm) channels using a 60x objective.

#### SARS-CoV-2 antibody detection

SARS-CoV-2 antibody levels were assessed in 63 PDGF samples of treated patients (13 severe cases; 50 moderate cases) using semiquantitative Ortho VITROS® Anti-SARS-CoV-2 Total (CoV2T) and Anti-SARS-CoV2-IgG (CoV2G) antibodies (Ortho Clinical Diagnostics, Raritan, New Jersey) and analyzed using the VITROS ECi/ECiQ 3600 Immunodiagnostic System following the manufacturer’s instructions. The results were reported as either reactive (S/CO ≥ 1.0) or nonreactive (S/CO < 1.0), and the S/CO was obtained using the manufacturer system [19].

#### PDGF metabolite measurement

We determined PDGF concentrations in 20 stem cells cocktail samples from COVID-19 patients. PDGF included: Angiopoietin-2 (Ang-2), epidermal growth factor (EGF), erythropoietin (EPO), fibroblast growth factor (FGF-basic), granulocyte-colony stimulation factor (G-CSF), granulocyte/macrophage-colony stimulation factor (GM-CSF), hepatocyte growth factor (HGF), macrophage-colony stimulation factor (M-CSF), platelet-derived growth factor AA (PDGF-AA), platelet-derived growth factor-BB (PDGF-BB), stem cell factor (SCF), T-cell growth factor-alpha (TGF-α), and vascular endothelial growth factor (VEGF). PDGF metabolite concentrations were determined using a 13-plex bead-based multiplex assay kit (LEGENDplex™ Human Growth Factor Panel; Cat. No. 740180) by fluorescence intensity in a Navios EX cytometer and analyzed with LEGENDplex V.8.0 software as recommended by the manufacturer.

### Treatment procedures and follow-up

PB-NHESC-C was delivered to the patients of Group A following compressor (jet) nebulization for a total of two doses 24 h apart. Both doses were administered through a sterile humidifier and a regular concentrated oxygen supply at a flow rate of 5–6 L/min. For clinical and laboratory evaluations, stem cell treatments were performed on days 0 and 1, and daily follow-up was performed for 28 days. Group B (control) was treated exclusively with the standard care provided to both groups.

All clinical, laboratory and radiological data were recorded during patient follow-up. From a clinical point of view, a detailed record included primary safety data (nebulization or standard treatment-induced allergic reactions, secondary infection, and severe and non-severe adverse events), and efficacy data were reported for the measurement of endpoints. Laboratory tests: a complete blood count determined using a Hematology Analyzer DHX900 (Beckman Coulter, USA) and acute phase reactants, such as C-reactive protein (CRP), which was assessed using a Chemistry Analyzer AU480 (Beckman Coulter, USA), and fibrinogen and D-dimer, which were assessed using a Cobas t 511 (Roche, Switzerland), according to the manufacturer’s instructions and in compliance with ADSCC standard operating procedures (SOP). SARS-CoV-2 RNA was assessed by real-time reverse transcription PCR before recruitment and after treatment [17]. Radiology: Both groups were followed using different imaging approaches, like X-rays and computer tomography scans (CT) until discharge, considering proposed recommendations for COVID-19 patients [20]. Forty CT were evaluated according to the critical criteria indicated using GE 46 Multi-Slice Computer Tomography (MSCT) (General Electric Healthcare System, USA).

### Outcomes evaluation

#### Primary safety data: adverse effects and disease assessment

Adverse events were assessed using the “Common Terminology Criteria for Adverse Events (CTCAE) v5.0” [21]. Group A patients were closely followed up for possible acute adverse events of treatment (AET) during the first 3 days after receiving stem cell nebulization. This follow-up continued for 28 days. AET in the control group was also evaluated during the same period.

#### Primary endpoints

- Hospital discharge. Assessed after the first 9 days of randomization: (1st. tertile of the follow-up).
- Mortality. Death by any cause in the 28 days of follow-up.

#### Secondary endpoints

- Accelerated clinical improvement after 9 days (1st. tertile of follow-up): Defined as a net decrease of at least 2 points on the scale (excluding patients who experienced increases in points in the period analyzed).
- Clinical improvement after 9 days (1st. tertile of follow-up). Defined as a net decrease of at least one point on the scale (excluding patients who experienced increases in points in the period analyzed).

#### Exploratory endpoints

- The persistence of lymphopenia.
- The appearance of lymphopenia.
- The persistence of an elevated neutrophil/lymphocyte ratio (NLR).
- The persistence of high C-reactive protein (CRP) levels.
- The persistence of elevated D-dimer levels (in the stem cell treated group).

All assessments were performed on the day of randomization and 5 days (1st quintile) after the intervention. Control group: evaluated on the day of randomization and 6 days later.

### Statistical analysis

Efficacy was assessed in the intention-to-treat and safety-as-treated groups in the study population. All statistical tests were performed to demonstrate the superiority of the assessed experimental therapy against the standard of care established for COVID-19. The Shapiro-Wilk test was performed to assess the normality of data distribution. The Mann-Whitney U non-parametric test was used for the analysis of two independent groups. Spearman’s rho correlation was applied in the comparison of downward changes in scores. The Wilcoxon test for non-parametric paired samples in laboratory variables before and after interventions as well as Fisher’s exact probability test for a small number of samples were also applied in the adverse events comparison, whereas the Chi-square test was used for categorical data, such as in the comparison of patient demographics and clinical proportions, adverse events and some laboratory variable analyses. To evaluate the clinical impact of interventions, the relative risk (RR) was calculated with a 95% confidence interval (CI), the relative risk reduction (RRR), and the number needed to treat (NNT) were also analyzed. Most of the statistical analyses were performed with GraphPad Prism v.8 (La Jolla, America) [22] and MedCalc software [23]. All *p*-values represented were two-sided and considered statistically significant when *p* < 0.05.

## Results

### Patient groups

Figure 1 shows the number of patients initially assessed for eligibility, the excluded ones, the randomized allocations into two groups, and patients receiving the different interventions and their follow-up and final analysis and outcomes. The main demographic and clinical status of the COVID-19 patients are shown in Table 1.

**Table 1:** Demographic data and clinical status of the enrolled patients on the recruitment day

Parameter	Group A: Treated with *PB-NHESC-C* plus standard care (*n* = 69)	Group B: Control (standard care only) (*n* = 70)	*p-*Value
**Demographic data**			
Age, years (mean ± SD)	45.93 ± 9.75	44.38 ± 11.09	0.380
Gender, n (%)			
Total	69 (48.94)	70 (51.06)	
Masculine	65 (46.10)	64 (46.81)	0.7447
Feminine	4 (2.88)	6 (4.32)	
Nationalities			
Afghanistan	2 (2.90)	1 (1.43)	0.6195
Bangladesh	11 (15.94)	11 (15.71)	1.0000
China PRP	0 (0)	1 (1.43)	1.0000
Egypt	3 (4.35)	3 (4.29)	1.0000
India	22 (31.88)	28 (40.00)	0.2920
Indonesia	1 (1.45)	0 (0)	0.4964
Jordan	0 (0)	1 (1.43)	1.0000
Nepal	5 (7.25)	2 (2.86)	0.2746
Pakistan	10 (14.49)	15 (21.43)	0.3777
Palestine	4 (5.80)	0 (0)	0.0581
Philippines	4 (5.80)	4 (5.71)	1.0000
Somalia	1 (1.45)	0 (0)	0.4964
Sudan	3 (4.35)	0 (0)	0.1196
Syria	1 (1.45)	2 (2.86)	1.0000
UAE	1 (1.45)	1 (1.43)	1.0000
USA	0 (0)	1 (1.43)	1.0000
Unknown	1 (1.45)	0 (0)	0.4964
**Health status at recruitment**			
Body mass index categories, n (%)			
Unknown	3 (4.35)	2 (2.86)	0.6806
Normal (healthy weight)	17 (24.64)	29 (41.43)	0.0472^a^
Overweight	35 (50.74)	19 (27.14)	0.0054^b^
Obese Class I (moderately obese)	11 (15.94)	13 (18.57)	0.8230
Obese Class II (severely obese)	0 (0)	3 (4.29)	0.2446
Obese Class III (very severely obese)	3 (4.35)	4 (5.71)	1.0000
Moderate COVID- 19, n (%)			
Score 3	37 (53.62)	40 (57.14)	0.7342
Score 4	12 (17.39)	6 (8.57)	0.1372
Subtotal	49 (71.01)	46 (65.71)	0.5852
Severe COVID-19, n (%)			
Score 5	3 (4.34)	7 (10.00)	0.3255
Score 6	2 (2.89)	1 (1.42)	0.6195
Score 7	15 (21.74)	16 (22.86)	1.0000
Subtotal	20 (28.98)	24 (34.28)	0.5852
Main comorbidities, n (%)			
Arterial hypertension	18 (26.09)	19 (27.14)	1.0000
Diabetes mellitus	18 (26.09)	13 (18.57)	0.3142
Cardiovascular disease and dyslipidemia	7 (6.25)	6 (8.57)	0.7792
Chronic smoking and asthma	11 (15.94)	4 (5.71)	0.0603

PB-NHESC-C: peripheral blood non-hematopoietic enriched stem cell cocktail; the *p*-value for age was determined by a χ^2^-test; the remaining *p*-values were determined by the F-exact probability test; ^a^: significant difference; ^b^: highly significant difference

Finally, 143 SARS CoV-2 confirmed by PCR patients were randomized, but only 139 were selected for the intervention (Fig. 1). Group A included 69 patients (65 males, four females) aged 45.93 ± 9.75 years old (mean ± SD; minimum 27, maximum 71) to assess the investigational product plus the standard COVID-19 care treatment established by the Ministry of Health and Prevention of the United Arab Emirates (MOHAP) [24]. Group B served as the control group with 70 patients (64 males, six females) aged 44.31 ± 11.22 years old (mean ± SD; minimum 26, maximum 73), recruited from the same hospitals, and receiving the standard care for COVID-19 exclusively. The standard care treatment for patients allocated to groups A and B included the following medication at baseline: Favipiravir, Lopinavir/Ritonavir or combined use of both drugs, as antivirals co-intervention in more than 82% of cases; Enoxaparin as an anticoagulant in 56% of cases; Tocilizumab as immunotherapy medications in 5% of cases; Hydroxychloroquine in the 89% of cases; and hydrocortisone in only three patients of group A as corticosteroids. In addition, antibiotics, antihypertensive and hypoglycemic drugs were applied in need cases.

The clinical score on the day of randomization was taken as a reference for the clinical follow-up. The composition of analyzed patients was 44 with severe disease: 31 critically ill (score 7), 13 severe patients (scores 5 and 6), and 95 moderate COVID-19 (score 3: *n* = 77, and score 4: *n* = 18). The conformation of the two arms for the trial was not statistically different in score and number of patients by Fisher’s exact probability test, as shown in Table 1.

### PB-NHESC-C characterization

The PB-NHESC-C was characterized by flow cytometry (Fig. 2A) and two main cell fractions with greater than 95% viability were noted: hematopoietic stem cells (CD45^dim^) in the range of 25–49% (median 36%) and non-hematopoietic stem cells (CD45^−^) in the range of 53–70% (median 64%) with one or more of the following markers: CD133^+^, CD90^+^ and CD34^+^. The PB-NHESC-C also contains anti-SARS-CoV-2 antibodies and PDGF. The median total number of nebulized cells was 2.2 ×  10^6^ for the whole group of treated patients, 1.8 × 10^6^ among severe COVID-19 patients, and 2.5 × 10^6^ in moderate cases. PB-NHESC-C was predominated comprised of CD45^−^/CD133^+^ and CD34^+^ cells. CD45^dim^ cell counts were significantly higher in moderate cases compared with severe ones (*p* < 0.0001), and the CD45^−^ cell count was higher in severe cases compared with moderate ones, but not statistically significant (*p* = 0.6). A positive correlation was observed between the patient’s absolute lymphocyte count and the number of CD45^dim^ cells in PB-NHESC-C (*p* = 0.006) and its absence with respect to CD45^−^ cells (*p* = 0.79). The CD45^−^/CD90^+^ marker predominated in the PB-NHESC-C of severe patients compared with moderate patients (*p* = 0.04), as shown in Table 2.

**Table 2:** Cellular and humoral components of the peripheral blood non-hematopoietic enriched stem cell cocktail

*Nebulized stem cell dose concerning severity in patients with coronavirus disease 2019 (COVID-19)*				
**Cellular elements, median (range)**	**Severe cases (*n*** **= 19)**	**Moderate cases (*n*** **= 50)**	**Total (*n*** **= 69)**	***p*-value**
Total stem cell dose (× 10^6^ cells)	1.8 (0.2–12)	2.5 (0.4–23)	2.2 (0.4–23)	0.34
Cellular CD markers (× 10^5^ cells)^b^				
CD45^−^	6.4 (0.1–49.5)	3.5 (0.1–7.1)	4.6 (0.1–7.1)	0.6081
CD45^dim^	0 (0–10.5)	5.2 (0.1–45.7)	3.7 (0–45.7)	< 0.0001^d^
CD133^+^	5.2 (1.1–31.2)	7.1 (0.2–69)	6.8 (0.2–69)	0.3056
CD34^+^	4.2 (0.2–37.1)	7.7 (0.1–52.7)	6.1 (0.1–52.7)	0.0920
CD90^+^	3.9 (0–13)	0.6 (0–12)	0.1 (0–13)	0.0485^c^
*The proportion of patients and semiquantitative values of anti-SARS-CoV-2 antibodies related to severity (S1 subunit of SARS-CoV-2 spike protein)*				
**Humoral factor**	**Severe cases**	**Moderate cases**	**Total evaluated**	***p*-value**
‘Patients’ anti-SARS-CoV2 antibodies, n (%)				
Total class antibodies	13 (100)	47 (94.0)	60 (95.2)	0.36
IgG class	11 (84.3)	24 (48.0)	35 (55.5)	0.018^c^
Optical density (OD) median value^b^	(*n* = 13)	(n = 50)	(*n* = 63)	
Total class antibodies	102.0	16.45	28.4	0.007^d^
IgG class	6.920	1.60	1.27	0.005^d^
*The concentration of platelet-derived growth factors in 20 selected COVID-19 patient samples of peripheral blood non-hematopoietic* *enriched stem cells cocktail (PB-NHESC-C)*				
**Platelet growth factor name (abbreviation)**			**Total evaluated (*n*** **= 20), pg/mL [median (range)]**	
Stem Cell Factor (SCF)			4.26 (4.26–21.51)	
Platelet Derived Growth Factor-AA (PDGF-AA)			382.3 (148.8–2542)	
Granulocyte-Colony Stimulation Factor (G-CSF)			18.28 (18.28–97.25)	
Hepatocyte Growth Factor (HGF)			25.91 (19.53–2885)	
Epidermal Growth Factor (EGF)			44.02 (4.40–125.2)	
Granulocyte Macrophage-Colony Stimulation Factor (GM-CSF)			8.99 (8.99–17.97)	
T-Cell Growth Factor alpha (TGF-ɑ)			1.61 (9.53–188.68)	
Platelet Derived Growth Factor-BB (PDGF-BB)			51.09 (16.04–187.2)	
Macrophage-Colony Stimulation Factor (M-CSF)			152 (73.69–268.8)	
Angiopoietin-2 (Ang-2)			35.51 (2.56–327.4)	
Fibroblast Growth Factor (FGF-basic)			1.6 (1.66–39.29)	
Vascular Endothelial Growth Factor (VEGF)			1.7 (8.72–91.96)	
Erythropoietin (EPO)			7.63 (14.46–65.55)	

^a^: *χ*^2^-test; ^b^: Mann-Whitney test for non-parametric samples; ^c^: significant difference; ^d^: highly significant difference; pg: picograms

ACE2 receptor expression was demonstrated in all CD45^−^ and CD45^dim^ cells (Fig. 2B). On the other hand, the presence of anti-SARS-CoV-2 total antibodies (COV2T) was detected in all cocktail samples of severe patients and 94% of moderate cases (*p* = 0.36). IgG anti-SARS-CoV-2 antibodies (COV2G) were more frequently observed (84.3%) in the autologous preparations of severe compared with moderate patients (48%) (*p* = 0.01). The semiquantitative values of total anti-CoV-2 (102.0 vs. 16.4, *p* = 0.007) and anti-anti-CoV-2 IgG (6.9 vs. 1.6, *p* = 0.005) were higher in severe cases than moderate cases. Therefore, the characterization of samples from PB-NHESC-C showed the presence of human PDGF derived from autologous PCP. PDGF-AA and M-SCF exhibited the highest concentrations, followed by PDGF-BB, EGF, Ang-2, and HGF, as shown in Table 2.

**Fig. 3:**
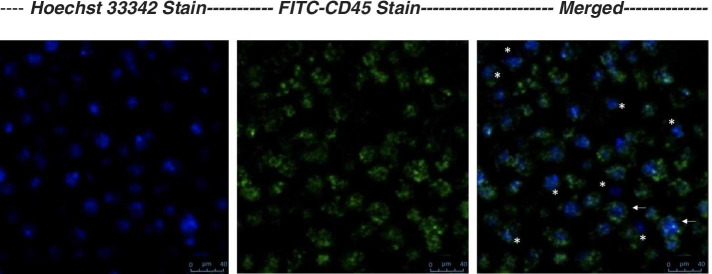
Representative immunofluorescence images of PB-NHESC-C. Legend: Sample of the PB-NHESC-C were stained with FITC-conjugated monoclonal surface antibody CD45 (1:100) and Hoechst nucleic acid dye 33,342 (10 μg/ml). Images were acquired using a Leica SP8 confocal microscope using a 63x objective. Two main subpopulations were identified: the Non-hematopoietic (*) and the Hematopoietic Stem Cells (arrow)

In addition, after the isolation of stem cells from the PB of patients, a representative image of PB-NHESC-C shows a slightly higher number of very small CD45 negative (CD45^−^) cells with a diameter of approximately 5–7 μm, compared with the fraction of cells dimly stained with CD45 (CD45^dim^) (Fig. 3).

### Outcomes

#### Primary safety data: assessment of adverse events and safety

Patients with adverse events were reported from both groups, with 101 (72.66% of enrolled patients) were affected. In total, 50 (72.46%) patients received stem cell treatment compared to 51 (72.85%) patients in the control group (*p* = 0.9419). A total of 240 adverse events were reported during the 28-day follow-up for all enrolled patients (Table 3)**.**

**Table 3:** Adverse events reported during the follow-up: SENTAD-COVID Study

Adverse events		PB-NHESC-C + standard care (***n*** = 69)	Standard care (***n*** = 70)	***p***-Value
Total patients affected by adverse events, n (%)		50 (72.46)	51 (72.85)	0.9590^a^
Total adverse events, n (%)		*n* = 240		
Adverse events by group, n (%)		107 (44.58)	133 (55.41)	0.8206^b^
Number of serious adverse events by group (including deaths)		35 (32.71)	57 (42.85)	0.1040^a^
**Adverse event type**	**Description**			
Total deaths, n (%)	Death	4 (5.79)	7 (10)	0.5319^c^
Other serious adverse events, n (%)				
Severe anemia	Hemoglobin reduction < 100 g/dL	10 (14.49)	8 (11.42)	0.5912 ^a^
Disease progression	Any score increased	5 (7.24)	9 (12.85)	0.3989^c^
Sepsis	Isolation of pathological fungal or bacterial from blood cultures indicating disseminated infections	5 (7.24)	15 (21.42)	0.0278*^c^
Acute renal failure	Hemodialysis of at least 2 days	4 (5.79)	6 (8.57)	0.7447^c^
Hypoxia	Oxygen saturation (SpO_2_) <88%	3 (4.34)	4 (5.71)	0.7184^c^
Acute respiratory distress syndrome	Respiratory rate >20 or <12 + SpO_2_ ≤ 88% + score ≥ 6	3 (4.34)	5 (7.14)	0.7184^c^
Multiorgan failure	Score changed to 7	1 (1.44)	3 (4.28)	0.6195^c^
Other nonserious adverse events				
Increased respiratory breath rate	O_2_ saturation levels ≥100	30 (43.47)	34 (48.57)	0.7348^a^
Hypertension	Blood pressure ≥ 120/80 mmHg	17 (24.63)	12 (17.14)	0.2789^a^
Fever	Temperature ≥ 38 °C	13 (18.84)	14 (20)	0.8633^a^
Severe decreased absolute lymphocyte count	Lymphocytes <0.8 × 10^9^/L	8 (11.59)	9 (12.85)	1.0000^a^
Sinus tachycardia	Heart rate (beats/min) ≥100	2 (2.89)	3 (4.28)	1.0000^c^
Sinus bradycardia	Heart rate (beats/min) < 60	1 (1.45)	2 (2.85)	1.0000^c^
Hypotension	Blood pressure < 100/60 mmHg	1 (1.45)	2 (2.85)	1.0000^c^

PB-NHESC-C: peripheral blood non-hematopoietic enriched stem cell cocktail, ^a^: χ^2^- test; ^b^: exact Poisson method; ^c^: Fisher’s exact test; ^*^: significant difference

Serious events, including patient death, were assessed. In total, 35 serious events occurred in the PB-NHESC-C-treated group, and 57 were noted in the control group.

#### Primary endpoints: PB-NHESC-C treatment partially demonstrated superior effects on hospital discharge and mortality reduction

##### Hospital discharge

After 9 days of follow-up (evaluating the first tertile after cell therapy), 63.3% of PB-NHESC-C-treated patients recovered and were discharged from hospitals. In the control group, this percentage was only 57.1%, but a nonsignificant difference was found. In addition, this patient’s hospital discharge after the first 9 days was related to a higher dose of CD45^dim^/CD34^+^ phenotype (1.4 × 10^6^ vs. 0, *p* = 0.0002) compared to the rest of non-discharged patients.

##### Mortality reduction

Although the outpoint regarding mortality reduction after 28 days of follow-up was not achieved, in the PB-NHESC-C treated group, the proportion of 5.8% death patients was lower than the 9.73% in the control group, but it was not statistically significant (*p* = 0.5319).

#### Secondary endpoints: clinical improvement over time due to PB-NHESC-C treatment observed in severe and moderate COVID-19 patients

**Fig. 4:**
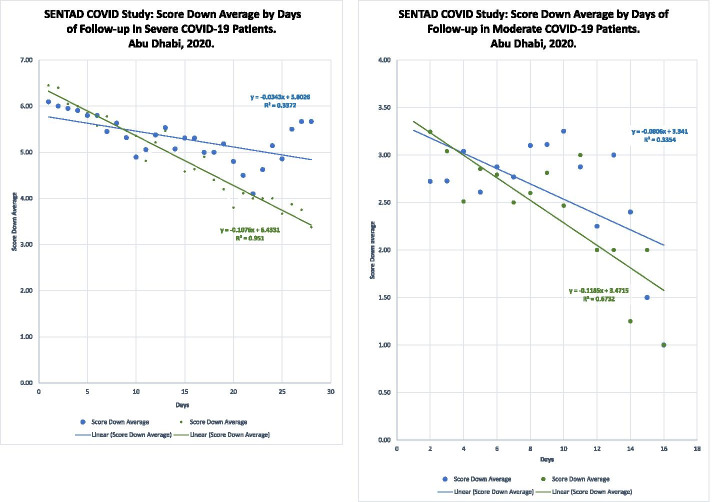
Clinical improvement. Different trend line slopes during the clinical trial follow-up. Legend: Group A/S: Peripheral Blood Non-Hematopoietic Enriched Stem Cell Cocktail (PB- NHESC-C) Treated classified as severe; Group B/S: Controls classified as severe; Group A/M: PB-NHESC-C Treated classified as moderate; Group B/M: Controls classified as moderate

From a clinical point of view, as seen in Fig. 4, severe COVID-19 patients treated with PB-NHESC-C resulted in more rapid clinical improvement than the corresponding control subgroup, as measured by the number of patients with clinical scores reductions daily. A trend line was noted in the score changes of this subgroup of stem cell-treated COVID-19 patients. The control evaluation yielded a highly significant difference in lines slopes between both groups (*p* ≤ 0.0001). Stem cell-treated moderate COVID-19 patients also improved and showed a linear trend compared with the equivalent controls with a significant difference in slopes, indicating better results for the stem cell-treated patients (*p* = 0.0074) during the first 15 days of the follow-up.

#### Clinical impact: the PB-NHESC-C-treated group exhibited a greater proportion of patients with clinical improvements

**Fig. 5:**
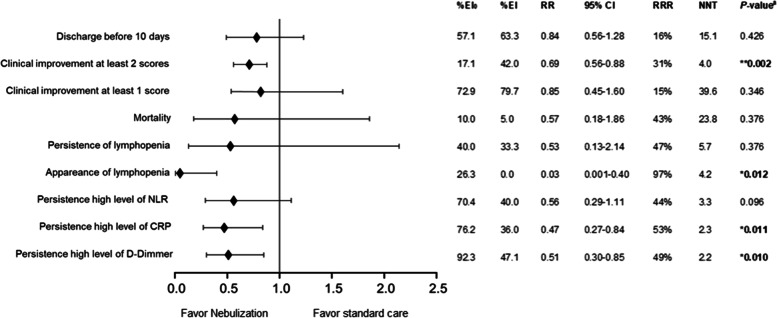
Clinical impact on the assessed outcomes. Legend: NLR: Neutrophil to Lymphocyte Ratio; CRP: C-Reactive Protein; EI_0_: Exposure Incidence in controls; EI: Exposure Incidence in peripheral blood non-hematopoietic enriched stem cell cocktail treated-patients; RR: relative risk; 95% CI: confidence interval; RRR: relative risk reduction; NNT: number needed to treat to produce the effect; ^a^: Z-test. *: significant; **: highly significant

A higher proportion of patients with clinical improvement was found in the PB-NHESC-C treated group than in the control group by assessing nine parameters at day 10 of treatment, as shown in Fig. 5. Statistical significance was seen for the proportions of clinical improvements in at least two scores between them (42.0% vs. 17.1%, RR = 0.85, *p* = 0.002), respectively. Furthermore, the RRR = 31% is another statistical fact demonstrated for this result. Therefore, if we were applying this type of stem cells therapy to the control group, the number of patients with this clinical improvement increased by 31%. The NNT showed a need to treat four patients with stem cells cocktail by nebulization to have one patient improvement in at least two scores in the first 9 days. Based on laboratory results, three other parameters also showed a statistically significant difference between both group: the appearance of lymphopenia (null vs. 26.3%, RR = 0.03; *p* = 0.012), the persistence of high levels of CRP (36.0% vs. 76.2%, RR = 0.47; *p* = 0.011), and of the D-dimer (47.1% vs. 92.3%, RR = 0.51; *p* = 0.010). All are considered indicators of COVID severity.

#### Exploratory endpoints: lymphocyte, neutrophils and acute phase reactant changes in PB-NHESC-C-treated patients

Table 4 shows the comparison of laboratory results before and after in each group of patients and the comparison between these groups after 28 days of follow-up.

**Table 4:** The laboratory follow-up of COVID-19 Patients During the Monitoring Period. Abu Dhabi, 2020

Variables	***PB-NHESC-C*** treated				Control			***p***-value^**b**^
	Time	n	Median (95% CI)	***p***-value^**a**^	n	Median (95% CI)	***p***-value^**a**^	
White blood cells (10^9^/L)	1st	51	6.8 (4, 20.45)	0.5377	35	6.855 (3.199, 13.45)	0.0753	0.0174*
	2nd		7.6 (3.84, 18.76)			10.99 (4.006, 24.5)		
Neutrophils (10^9^/L)	1st	51	4.4 (1.6, 17.75)	0.2251	35	4.49 (1.812, 12)	0.3023	0.0029**
	2nd		4.3 (1.5, 15.18)			7.27 (2.21, 22.65)		
Lymphocytes (10^9^/L)	1st	51	1.305 (0.55, 3.2)	< 0.0001***	35	1.305 (0.5205, 3.46)	0.068	0.0017**
	2nd		2.1 (0.802, 3.38)			1.64 (0.38, 2.758)		
Monocytes (10^9^/L)	1st	51	0.6 (0.3, 1.25)	0.9838	35	0.535 (0.1355, 1.078)	0.0017**	0.019*
	2nd		0.6 (0.3, 1.504)			0.88 (0.302, 1.852)		
Eosinophils (10^9^/L)	1st	51	0.1 (0, 0.3)	< 0.0001***	35	0.075 (0.00, 0.4155)	0.0074**	0.3378
	2nd		0.2 (0, 0.82)			0.18 (0.00, 0.81)		
Basophils (10^9^/L)	1st	51	0.0 (0, 0.1)	< 0.0001***	35	0.02 (0.00, 0.0645)	0.0109*	0.3925
	2nd		0.1 (0, 0.2)			0.03 (0.00, 0.158)		
NLR (U)	1st	51	3 (0.86, 15.66)	0.0011**	35	2.6 (0.84, 15.7)	0.5764	< 0.0001***
	2nd		1.8 (0.56, 16.79)			4.3 (1.1, 57.66)		
D-Dimer (μg FEU/mL)	1st	49	0.4 (0.2, 8.1)	0.1113	31	0.57 (0.21, 14.8)	0.3931	< 0.0001***
	2nd		0.2 (0, 3.6)			1.51 (0.26, 537.7)		
Fibrinogen (g/dL)	1st	31	718 (380, 1136)	< 0.0001***	13	420 (240, 1940)	0.0625	< 0.0001***
	2nd		350 (220, 69)			470 (390, 980)		
IL-6 (pg/mL)	1st	31	20.3 (1.5, 3361)	< 0.0001***	0	ND	ND	ND
	2nd		3.05 (1.4, 3384)			ND		
C-reactive protein (mg/L)	1st	31	34 (0.6, 381.8)	< 0.0001***	24	21.79 (0.4, 335.3)	0.0049**	0.0048**
	2nd		3.6 (0.42, 111)			16.45 (0.6, 350)		

Time: comparing 1st and 2nd studies inside each group; n: sample size; NLR: neutrophil/lymphocyte ratio; ND: not determined; 95% CI: Confidence interval; ^a^: Wilcoxon test for nonparametric pair samples; ^b^: Mann-Whitney U test, comparing the two groups; *: significant difference; **: higly significant; ***: very higly significant

After treatment with PB-NHESC-C, all COVID-19 patients showed better results in normalizing the absolute number of lymphocytes and improving the neutrophil/lymphocyte ratio compared with the control group. In addition, fibrinogen, IL-6, and C-reactive protein levels also significantly decreased in the treated group during the follow-up. In the control group, only statistically significant changes were observed in the reduction of C-reactive protein levels. Comparing blood cell counts in both groups, differences were observed in the levels of absolute white blood cells, absolute lymphocytes, neutrophils, monocytes, the neutrophils/lymphocyte ratio, and acute phase reactants, such as D-dimer, fibrinogen, and C-reactive protein, because the changes in the controls were not as notable as those in the PB-NHESC-C-treated patients.

#### High-resolution computer tomography scans (CT) images of a Patient’s chest

**Fig. 6:**
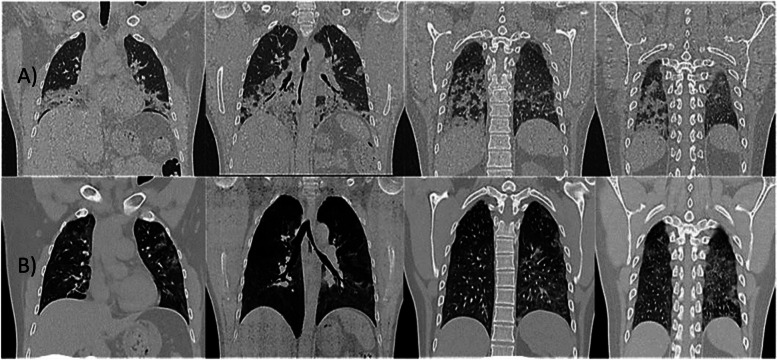
High-Resolution Computer Tomography Scans Images of a Patient’s Chest. Legend: Patient No. 4. Group A (peripheral blood non-hematopoietic enriched stem cell cocktail + standard care): Images a) Day of recruitment (April 1st). Images b) Four days after the first dose of stem cell treatment (April 13th, nebulization was initiated on April 9th, and the second dose was commenced on April 10th)

The evaluated CT corresponds to 37 patients from group A and three from group B. Nevertheless, Fig. 6 shown representative CT images of one group PB-NFESC-C treated COVID severe patient with a score of 4 as an example of his fast recovery.

On the day of recruitment, patient No. 4 CT chest images showed patchy consolidations in the bilateral lower lobes and right middle lobe as well as few peripheral consolidations in the left upper lobe and patchy peripheral ground-glass opacities in the upper lobes. An important change in improvement was noted after 4 days of nebulization.

## Discussion

The clinical data of recruited patients at the time of inclusion included all signs and symptoms that were more frequently noted in COVID-19 patients, including fever, weakness, shortness of breath, secondary bacterial sepsis, and lower oxygen saturation [25].

The loss of patients during the follow-up was the main limitation for collecting data because many of the patients in the first and second tertiles recovered and were released from hospitals, rendering some statistical limitations and bias in this study.

In addition, the quantities of each allocation group changed daily, so data monitoring was performed to evaluate clinical improvements and adverse events, including deaths. However, a proper quantitative meta-analysis, with a larger sample could be designed to validate this study and better assess the effect of treatment.

The randomization yielded 69 patients for Group A and 70 for Group B. Age, gender, nationalities, and other demographic and clinical information obtained at the time of recruitment were similar between both groups, and the only significant difference was found in a portion of body mass index (BMI) categories, such as normal weight and overweight. For both of these factors, the control group exhibited better values. A smaller number of patients with these disadvantages were included by chance in the stem cell-treated group and some reports have noted a poor prognosis for overweight patients [26].The short number of female patients in the trial may be related to the economically active resident population in UAE, characterized by expatriate young male workers, most of them living in only-male facility residences without their family in this country. Patients in the trial represent 15 nationalities, which is similar to that noted in the UAE population, giving strong meaning to the study.

This work describes the characterization of a cocktail of autologous stem cells suspended in PDGF that also includes anti-SARS-CoV-2 antibodies. These cells express markers CD133 and CD34, known as pluripotent markers, and to a lesser extent CD90; the cocktail has a similar number of circulating endothelial cells to that reported in response to endothelial damage and dysfunction in many diseases [27]. The CD45^−^ cell fraction is similar in size and phenotype to that described for VSELs, but further studies will be needed for better cell characterization [28]. Given that PB-NHESC-C is a product obtained from COVID-19 patients, the phenotypes cellular number and variability is influenced by the immune response to viral infection and the mobilization of progenitor cells to damaged tissue. Therefore, these differences explain what we found in this study between the severe and moderate groups [29]. Indeed, the significant CD5^dim^ cell count in the cellular preparation of moderate cases is synergically correlated with the absolute count of lymphocytes in peripheral blood, indicating their potential role as lymphocyte precursors. The increase in CD45^−^ and CD90^+^ cell counts found in the PB-NHESC-C of severe patients could be representing an increase in a VSELs mobilization under stress conditions in response to the release of inflammatory cytokines, which is known as one of the red flags of the disease [30].

The effects of stem cell therapy are dose dependent. The efficacy of the stem cell cocktail product should be evaluated based on the presence, phenotype, and function of different cellular subsets. Nevertheless, in this work we didn’t find a relationship between the total dose of stem cells administered and the product’s efficacy. Besides, our results were more correlated with specific stem cell subsets. Indeed, hospital discharge before 10 days was related to the number of CD45^dim^ cells and, more specifically, CD45^dim^/CD34^+^ cells.

Furthermore, in spite of the well-known role of CD34^+^ hematopoietic stem cells in hematopoietic transplantation [31], their therapeutic role in pulmonary diseases has not been widely studied. A few supportive studies have reported their increase in PB during interstitial lung disease as a compensatory mechanism of tissue repair [32] and their ability to differentiate into other cell lineages, including epithelial cells [33]. CD34 is expressed in a wide range of cells in addition to hematopoietic cells and stromal, epithelial, and endothelial cells. The function of this marker has not been fully clarified, but it is associated with the inhibition or facilitation of adhesion, proliferation, and regulation of cell differentiation [34]. A significant increase in the PB of CD45^dim^/CD34^+^ cells in severe COVID^−^ 19 patients was reported [29] and interpreted as a response to endothelial regeneration during hypoxia. No relationship was found between the number of CD34^+^ cells in PB-NHESC-C and disease severity in our study. These contradictory findings may be due to differences in study populations, methodology, and parameter analysis.

Furthermore, we found that nebulization of a high dose of CD90^+^ cells was associated with a longer hospital stay, and CD90^+^ cells were more highly expressed in PB-NHESC-C cells of severe patients who required a longer hospital stay. This finding was not expected given the immunosuppressive activity of CD90 that controls inflammation. We attributed the alteration of CD90 activity to the high expression of proinflammatory cytokines, such as IL-1β and TNFα, which are commonly increased in COVID-19 patients [35].

On the other hand, it is well known that ACE2 is expressed in most human cells, and SARS-CoV-2 enters the host cell via binding of the S protein on the viral surface to ACE2 on the cell surface [36]. In addition to the lung, ACE2 is widely expressed in human tissues, including the heart, liver, kidney, and digestive organs [37]. Almost all endothelial cells and smooth muscle cells in organs express ACE2; therefore, we measured ACE2 expression in our investigational product. A recent study demonstrated that ACE2 and the entry-facilitating transmembrane protease TMPRSS2 are expressed on VSELs, and it is hypothesized that the interaction of its receptor activates the Nlrp3 inflammasome. Thus, hyperactivation of these cells can promote the death of infected cells by pyroptosis [38]. Nevertheless, there was no evidence of the loss of viability in the stem cell cocktail in this trial, and SARS-CoV-2 did not damage the cells. If the cells were destroyed, the PB-NHESC-C cocktail would not demonstrate efficacy in controlling the patient’s cytokine storm.

Positivity for anti-SARS-CoV-2 IgG antibodies and the semiquantitative estimation of anti-SARS-CoV-2 T/G did not show beneficial effects on endpoints. Antibody nebulization of human plasma does not cause loss of immunoglobulin function in animal models [39] and is well tolerated for human treatment [40]. Nevertheless, the exclusive presence of anti-SARS-CoV-2 IgG antibodies in the nebulized product evaluated in this study also potentially influenced the effectiveness of PB-NHESC-C therapy despite not being related to antibody concentration. This notion should be clarified in other studies, and these results could be influenced because the concentration of anti-SARS-CoV-2 antibodies was significantly higher in the group of severe patients who require a longer hospital stay due to disease severity.

Additionally, we evaluated the presence of human growth factor in PDGF, which was used as an excipient of the stem cell cocktail, among a small number of cases with the purpose of better characterizing the final investigational product. Notably, it was not possible to relate these factors with efficacy. In previous communications, similar profiles of angiogenesis and endothelial damage markers in the peripheral blood of patients were obtained in noncritical and critical phases of COVID-19 [41]. Higher concentrations of factors, such as PDGF-AA and M-SCF, play an essential role in many processes related to the immune response, angiogenesis, and tissue repair [42]. A potential role of PDGF and its contribution to the efficacy of the treatment was not addressed in this study and is currently under further investigation.

Death, sepsis, disease progression, acute renal failure, hypoxia, acute respiratory distress syndrome, and multiorgan failure are serious adverse events [41]. However, their frequencies were lower in group A than in group B. Only the increased sepsis frequency found in the control group was significantly different between the two groups. It is known that SARS-CoV-2 infection predisposes people to have bacterial and fungal sepsis complications or even a septic shock. In addition, it is associated with high mortality of severe COVID-19 patients. Therefore, this difference can be assumed as a protective effect of the investigational product not observed in the control group. Severe anemia, an expected adverse event after blood collection for the PB-NHESC-C preparation, was similar in both groups, indicating a correlation with SARS-CoV-2 infection and not with the treatment because anemia has also been reported a COVID-19 complication [43, 44]. None of the considered nonserious events assessed, such as increased respiratory breath rate, hypertension, fever, decreased absolute lymphocyte count, tachycardia, bradycardia, and hypotension, showed significant differences between the groups. In addition, administration-related or acute allergic reactions were not observed within 2 h to 3 days after nebulization. Therefore, the use of PB-NHESC-C didn’t lead to delayed hypersensitivity or secondary infections in 28 days of follow-up.

The clinical improvement observed in severe and moderate COVID-19 patients during the follow-up supported the potential application of stem cell cocktails. The trend line slope in both patients treated with stem cells showed a better adjustment to the right line and less dispersion to the center compared with patients treated with the standard of care, indicating that PB-NHESC-C facilitates accelerated clinical improvement that portend an improved recovery prognosis compared with those treated with standard care alone. However, no relationship was found between a reduction of 2 or more points on the disease severity scale and the dose of nebulized cells, cell phenotypes, or positivity for anti-SARS-CoV2, total and IgG antibodies.

Some reports also found the appearance of lymphopenia as well as high levels of D-dimer and C-reactive protein in COVID-19 patients [45, 46]. In general, the elevation of D-dimer levels is not a specific response and is most commonly used in the diagnosis of venous thromboembolism, pulmonary embolism, and acute abdomen disorders [47, 48]. C-reactive protein is a biomarker with high sensitivity for inflammation. It is part of the host response to the production of cytokines, particularly TNFα, IL-6, MCP1, and IL-8, which are secreted by several immune cells, including T cells. An increased C-reactive protein levels are also indicative of a myocardial effect [49].

Therapy with compressor-nebulized PB-NHESC-C modifies the immune response as demonstrated by statistically significant changes in acute phase serum markers and coagulation tests, such as D-dimer, after treatment in COVID-19 patients. As found in this study, the levels of some acute phase reactants in COVID-19 patients were higher than the normal range at the start of stem cell therapy. Hence, the cytokine release syndrome caused by abnormally activated immune cells resulted in deterioration of the patient’s condition, which may alter endothelial cell function, induce capillary leakage, promote mucus blockage in the lung and induce respiratory failure. These effects could even cause an inflammatory cytokine storm leading to multiple organ failure. However, this effect was reverted in most patients when their levels of inflammatory reactants were normalized after treatment with PB-NHESC-C.

Various chest imaging features have been reported in COVID-19, and the images are similar to those found in other types of coronavirus syndromes. However, in this study, the CT chest image provides an example of a patient who rapidly improved after investigational product treatment.

PB-NHESC-C cocktail therapy may inhibit the over-activation of the immune system and significantly improve inflammation even in severe COVID-19 patients. The majority of patients with severe COVID-19 pneumonia survive and recover. The nebulization of PB-NHESC-C improved the outcome of COVID-19 patients may be due to regulation of the inflammatory response and the promotion of tissue repair and regeneration. However, a statistically significant difference in the percentage of patients discharged from the hospital was not noted. After day 9, the value was higher in the PB-NHESC-C treated group compared with controls with a clinical impact of RR = 0.84.

Additionally, the proportion of patients with an improved NLR in the stem cell-treated group was greater than that in the controls. Both clinical impact factors favored nebulization, as NLR levels have been a predictive marker for severity and mortality in COVID-19 [50]. More improvements measured by at least a 2-point reduction on the disease severity scale were noted in the treated group with a highly statistically significant difference. In contrast, more improvements measured a 1-point drop were pointed out in the treated group, but these results were not significant. However, it should be noted that treatment does not yield equivalent or inferior effects. Increasing the size of the sample could lead to better statistically significant differences in results.

The cure for COVID-19 is essentially dependent on the patient’s immune system. When the over-activated immune system kills the virus, it produces many inflammatory factors, leading to severe cytokine storms, and older patients may be more easily affected due to immunosenescence [51]. Cells, such as mesenchymal stem cells, have been tested to treat lung diseases using nebulization and aerosols [52, 53]. In cases of COVID-19, other cellular therapy products using different types of stem cells have also aided patient’s recovery [13, 54–56].

## Conclusions

This paper is the first report of an autologous treatment with minimally manipulated stem cells. The main component of the cocktail is non-hematopoietic cells, which were obtained using a simplified autologous cell isolation procedure that can be implemented in blood banks or transfusion center facilities. The PB-NHESC-C was safe and improved the clinical and laboratory outcomes in most treated patients with the potential to reduce hospitalization and mortality.

## Data Availability

Datasets used and/or analyzed during the current study are available from the corresponding author on reasonable request.

## Abbreviations

AAD: Amino-actinomycin D
ACE2: Angiotensin-converting enzyme 2
ADSCC: Abu Dhabi Stem Cells Center
Ang-2: Angiopoietin-2
APC: Allophycocyanin
ARDS: Acute Respiratory Distress Syndrome
CTCAE: Common Terminology Criteria for Adverse Events
COVID-19: Coronavirus disease 2019
CoV2T: SARS-CoV2 total antibodies
CoV2G: SARS-CoV2 IgG antibodies
CI: Confidence interval
CT: Computerized tomography scans
EGF: Epidermal growth factor
EPO: Erythropoietin
FGF: Fibroblast growth factor
FICT: Fluorescein isothiocyanate
G-CSF: Granulocyte-colony stimulation factor
GM-CSF: Granulocyte/macrophage-colony stimulation factor
HSCs: Hematopoietic stem cells
HGF: Hepatocyte growth factor
HBV: Hepatitis B virus
HIV: Human immunodeficiency virus
iPSCs: Induced pluripotent stem cells
IRB: Institutional review board
M-CSF: Macrophage-colony stimulation factor
MSCs: Mesenchymal stem cells
NLR: Neutrophil to lymphocyte ratio
NNT: Number needed to be treated
PB: Peripheral blood
PB-NHESC-C: Peripheral blood-derived non-hematopoietic enriched stem cell cocktail
PDGF-AA: Platelet-derived growth factor AA
PDGF-BB: Platelet-derived growth factor BB
PDGF: Platelet-derived growth factors
PCP: Platelet-concentrated plasma
REC: Research Ethics Committee
RNA: Ribonucleic acid
RR: Relative risk
RRR: Relative risk reduction
RT: Room temperature
RT-PCR: Real-time Polymerase Chain Reaction
SARS-CoV-2: Severe Acute Respiratory Syndrome Coronavirus-2
SCF: Stem cell factor
SpO_2_: Oxygen Saturation
TGF-α: T-cell growth factor-alpha
TMPRSS2: Transmembrane protease-serine 2
VSELs: Very small embryonic-like stem cells
VEGF: Vascular endothelial growth factor
WHO: World Health Organization
